# Organotypic slice cultures of human gastric and esophagogastric junction cancer

**DOI:** 10.1002/cam4.720

**Published:** 2016-04-12

**Authors:** Justus Koerfer, Sonja Kallendrusch, Felicitas Merz, Christian Wittekind, Christoph Kubick, Woubet T. Kassahun, Guido Schumacher, Christian Moebius, Nikolaus Gaßler, Nikolas Schopow, Daniela Geister, Volker Wiechmann, Arved Weimann, Christian Eckmann, Achim Aigner, Ingo Bechmann, Florian Lordick

**Affiliations:** ^1^ Institute for AnatomyUniversity Medicine LeipzigLiebigstraße 1304103LeipzigGermany; ^2^ University Cancer Center Leipzig (UCCL)University Medicine LeipzigLiebigstraße 2004103LeipzigGermany; ^3^ Institute of PathologyUniversity Medicine LeipzigLiebigstraße 2404103LeipzigGermany; ^4^ Department for Visceral, Transplantation Thoracic and Vascular SurgeryUniversity Medicine LeipzigLiebigstraße 2004103LeipzigGermany; ^5^ Department for General and Visceral SurgeryKlinikum BraunschweigSalzdahlumer Straße 9038126BraunschweigGermany; ^6^ Institute of PathologyKlinikum BraunschweigCeller Straße 3838114BraunschweigGermany; ^7^ Institute of PathologyKlinikum St. GeorgDelitzscher Str. 14104129LeipzigGermany; ^8^ Department for General and Visceral SurgeryKlinikum St. GeorgDelitzscher Str. 14104129LeipzigGermany; ^9^ Department for General, Visceral and Thoracic SurgeryKlinikum PeineVirchowstraße 831226PeineGermany; ^10^ Rudolf‐Boehm‐Institute for Pharmacology and ToxicologyClinical PharmacologyUniversity Medicine LeipzigHärtelstraße 16‐1804107LeipzigGermany

**Keywords:** Chemosensitivity, esophagogastric junction cancer, gastric cancer, organotypic slice cultures, personalized treatment

## Abstract

Gastric and esophagogastric junction cancers are heterogeneous and aggressive tumors with an unpredictable response to cytotoxic treatment. New methods allowing for the analysis of drug resistance are needed. Here, we describe a novel technique by which human tumor specimens can be cultured ex vivo*,* preserving parts of the natural cancer microenvironment. Using a tissue chopper, fresh surgical tissue samples were cut in 400 *μ*m slices and cultivated in 6‐well plates for up to 6 days. The slices were processed for routine histopathology and immunohistochemistry. Cytokeratin stains (CK8, AE1/3) were applied for determining tumor cellularity, Ki‐67 for proliferation, and cleaved caspase‐3 staining for apoptosis. The slices were analyzed under naive conditions and following 2–4 days in vitro exposure to 5‐FU and cisplatin. The slice culture technology allowed for a good preservation of tissue morphology and tumor cell integrity during the culture period. After chemotherapy exposure, a loss of tumor cellularity and an increase in apoptosis were observed. Drug sensitivity of the tumors could be assessed. Organotypic slice cultures of gastric and esophagogastric junction cancers were successfully established. Cytotoxic drug effects could be monitored. They may be used to examine mechanisms of drug resistance in human tissue and may provide a unique and powerful ex vivo platform for the prediction of treatment response.

## Introduction

Gastric cancer (GC) and adenocarcinomas of the esophagogastric junction (AEG) are aggressive and highly heterogeneous tumors 1. Due to their critical prognosis following curative resection (R0), adjuvant treatment is the standard of care in most parts of the world. Western world studies advise perioperative chemotherapy or chemoradiation 2, 3, 4, 5, whereas postoperative chemotherapy is the standard of care in East Asia 6, 7, 8. The preferred chemotherapy regimens are based on fluoropyrimidines and platinum compounds which may be combined with epirubicin or docetaxel 1, 8.

GC and AEG have unpredictable responses to cytotoxic treatment and the majority of patients are nonresponders 9, 10. Nonresponding patients receive toxic treatment with no benefit or even detrimental effects on outcomes 11, 12, 13, 14. The identification of nonresponders before starting perioperative therapy would be of utmost importance. New methods are needed to overcome the limitations of established models in predicting treatment response 15.

Recent studies from our laboratory demonstrated that human cancers can be brought into organotypic slice cultures 16, 17. This novel method enables to study cytotoxic drug and ionizing radiation effects on individual human cancer probes. A major advantage of this model in contrast to cell culture and cell culture‐derived xenograft experiments is the partial preservation of the human tumor microenvironment 16, 17, 18, 19. However, different tumor entities need to be established on its own, as they display distinct growth and culture characteristics, and the complexity of tumor tissue requires careful investigation. For the first time, we could culture tissue slices derived from human GC and AEG and demonstrate their potential for assessment of cytotoxic drug efficacy.

## Material and Methods

### Preparation and experimental set‐up

Fresh surgically sampled human tissue of GC and AEG were obtained after pathological routine diagnostics. Patients were chemonaive or had undergone neoadjuvant chemotherapy. Tumor samples were transported in culture media and were further processed within 4 to 24 h postextraction. Under sterile conditions, surgical tumor specimens were dissected with an autoclaved razor blade in pieces of a height of 0.5 cm for further preparation. These pieces were cut in 400 *μ*m thick slices using a tissue chopper (McIlwain TC 752; Campden Instruments, Lafayette, IL) and were carefully separated under a stereo microscope with forceps and a scalpel. Then, tissue slices were placed on membrane inserts (Millipore Corporation, Billerica, MA) and were cultured in 6‐well plates. Each well contained 1 ml culture medium under the membrane inserts supplying the tissue via diffusion. RPMI‐1640 (Gibco, Life technologies, Paisly, UK) culture medium was supplemented with 10% fetal calf serum (Fetal Calf Serum [FCS]; Invitrogen, Darmstadt, Germany), 1% l‐glutamine (Gibco), 1% amphotericin B (Carl Roth, Karlsruhe, Germany), and 1% penicillin/streptomycin (Gibco). Slices were incubated in a humidified incubator at 37°C and 5% CO_2_ for 2, 4, and 6 days. The culture medium was changed every second day. Slices, which were fixed at the preparation day, were labeled as day 0. Each culture time point was compared with day 0 and is represented by an individual slice.

The study was approved by the ethics committee of the University of Leipzig Medical Faculty. All patients who donated tissue declared their informed consent in written form.

### Chemotherapy exposure

Slices were incubated with 5‐FU (Medac, Wedel, Germany) and cisplatin (Neocorp, Weilheim, Germany) at different concentrations immediately after cultivation. Drugs were dissolved in 0.9% NaCl and diluted in the culture medium to final concentrations of 3, 10, and 100 *μ*mol/L for 5‐FU and 3, 10, and 30 *μ*mol/L for cisplatin. Slices were exposed to cytotoxic treatment over 2–4 days. Drug containing culture medium was freshly prepared before application and changed every other day. Slices of the same tumor that were not exposed to cytotoxic drugs but cultured and fixed simultaneously served as controls.

### Staining

Tissue slices were fixed in 4% paraformaldehyde overnight, embedded in paraffin for standard histomorphology using hematoxylin–eosin (H&E) and periodic acid–Schiff (PAS). Cytokeratin stains (CK8 antibody; BioGenex, Fremont, CA: mouse, 1:100 and AE1/3 antibody (CK1‐8, 10, 14‐16, and 19) are labeled as CK in figures; BioGenex, mouse, 1:50) were used for determining tumor cellularity, Ki‐67 (DCS Innovative Diagnostik‐Systeme, Hamburg, Germany: rabbit, 1:400) for proliferation, and cleaved caspase‐3 staining (Cell Signaling Technology, Danvers, MA: rabbit, 1:400) for apoptosis. CK‐positive cells were defined as tumor cells if normal gastric mucosa could not be detected in the H&E stain. A double staining of CK/Ki‐67 and CK/cleaved caspase‐3 was performed to define the tumor cell viability. After citrate buffer treatment at 95°C for 10 min, sections were washed in 1.5% Triton/PBS, blocked with 5% normal goat serum for 30 min, and incubated overnight at 4°C with primary antibodies. After rinsing sections repeatedly with 1.5% Triton/PBS, primary antibodies were coupled with fluorescent‐labeled secondary antibodies (goat‐anti‐mouse 568, goat‐anti‐rabbit 488, Alexa Fluor, Invitrogen, Eugene, OR) and nuclei were counterstained with Hoechst 33342 (Sigma, St. Louis, MO).

### Analysis of slices

To determine the viability of tissue slices, stained slides were examined under an Olympus BX51 fluorescent microscope (Olympus Deutschland, Hamburg, Germany). Total cell number (Hoechst‐positive nuclei) was counted by the ImageJ cell counter plugin (NIH, Bethesda, MD). CK, cleaved caspase‐3, and Ki‐67‐positive cells were counted manually. These three parameters were put in relation to the total cell number and defined as the tumor cell fraction, the apoptosis, and proliferation indices. For determining total cell number and tumor cellularity, six pictures (*n* = 6) in a 200× magnification per group were taken and counted. Three pictures (*n* = 3) in a 400× magnification per group were counted for determining apoptosis and proliferation indices. Statistics were accomplished using GraphPad Prism 6 (GraphPad Software, Inc., La Jolla, CA). One‐way analysis of variance (ANOVA) was used and *P* < 0.05 was considered to be statistically significant.

## Results

### Manufacturing and analysis of slice cultures

**Figure 1:**
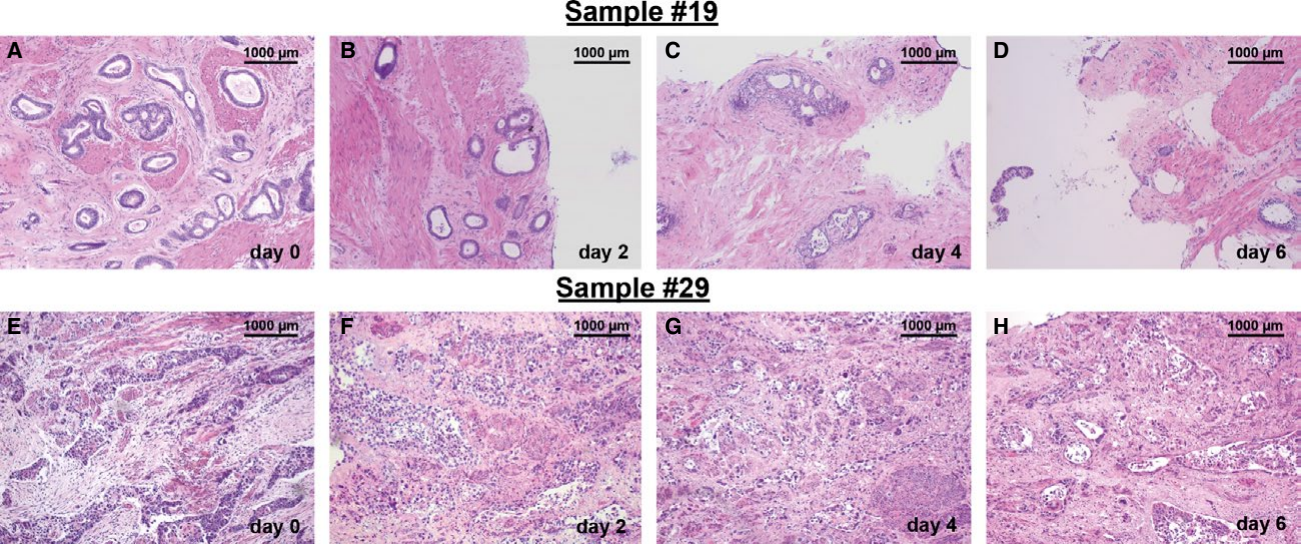
Overview of histopathology of cultured tissue slices derived from GC and AEG surgical specimen. Tissue slices derived from GC (A–D) and AEG (E–H) surgical specimens were cut in 400 *μ*m thick slices and kept ex vivo over 2, 4, and 6 days. Slices were processed to paraffin sections (7 *μ*m) and stained with H&E. Low magnification pictures show an overview of cultured tissue. Initial gland structures and tissue morphology can be observed also at day 2, 4, and 6 in vitro (A–D). Density of glands is reduced at day 6 (D). Original magnification: 100× in (A–H).

**Figure 2:**
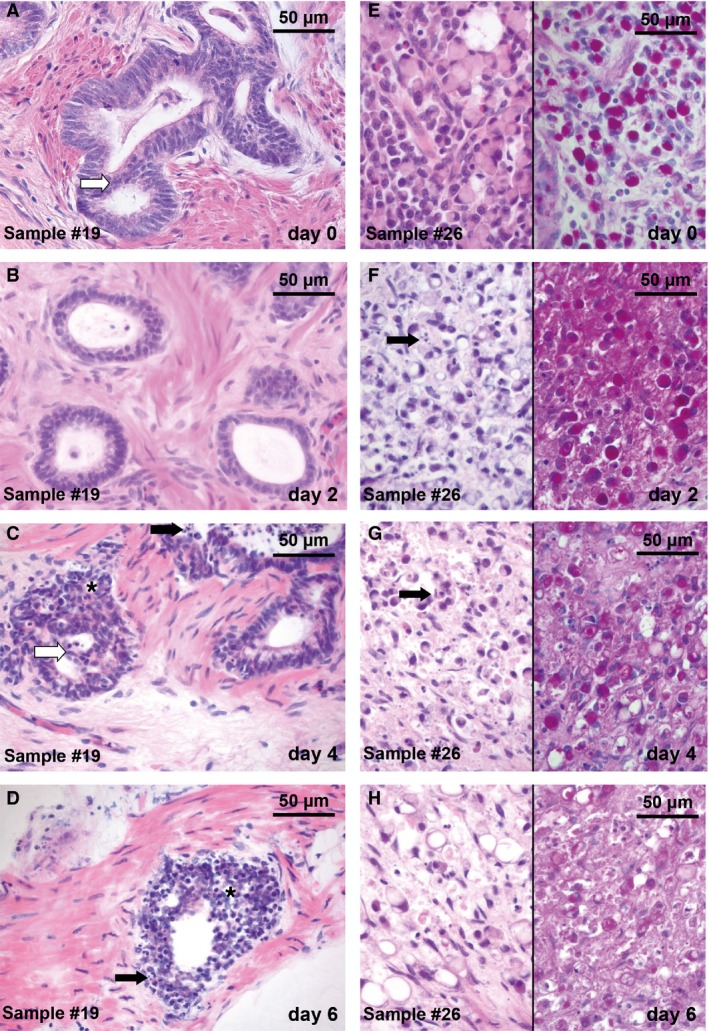
Histopathology of cultured tissue slices derived from GC surgical specimen. Tissue was cut in 400 *μ*m thick slices and kept ex vivo over 2, 4, and 6 days. Slices were processed to paraffin sections (7 *μ*m) and stained with H&E (A–D and E–H, left column) and PAS (E–H, right column). Two different tumor samples derived from surgical specimens are shown: intestinal subtype (A–D) and diffuse subtype (E–H). Slice cultures revealed a good preservation of tissue morphology and tumor cell integrity compared with day 0. (A) Gland‐forming structures, pleomorphic nuclei, and a shifted nuclear/cytoplasmic ratio characterize the intestinal subtype. (B–D) These features persisted during the culture period up to day 4 in vitro. Intraluminal bridges (A; white arrow) and a change of gland formation structures (C, D; *) were observed. Apoptotic bodies (C, D, F, G; black arrow) were detectable during cultivation. (E–H) Mucin‐containing signet‐ring cells (pink stain in PAS) signify the diffuse subtype. Morphology of signet ring cells remained stable (G) until day 4 in vitro. Original magnification: 400× in (A–H).

Tissue was obtained from 13 patients after resection (nine GCs and four AEGs). Eight cases were used for qualitative and quantitative analysis (see Table S1). In five samples, analysis of slice cultures was not possible due to sparse tumor in the tissue sample (two cases), insufficient interslice comparability (two cases), or fungal infection (one case). A topographical orientation of the surgical tissue sample was needed to trim the samples accurately before slicing. Furthermore, the precise collection of consecutive slices was important to allow for a good interslice comparison within consecutive series. Taking this into account, slice cultures revealed a good preservation of tissue morphology and cellular integrity up to 6 days. GCs of the intestinal and diffuse subtype generally maintained a stable tumor cellularity and stroma preservation (Figs. 1 and 2).

No major difference in cellularity between upper and lower layers could be distinguished in vertical H&E‐stained sections (Fig. S1A, B), ruling out a vertical viability gradient caused by diffusion of nutrients from the bottom of the cultures. In line with this observation, induction of apoptosis by 5‐FU treatment was also observed along the entire vertical diameter, proving the equal accessibility of cells within the slice for medium components as well as cytotoxic drugs (Fig. S1C and D).

### Quantification of stability of slice cultures

**Figure 3:**
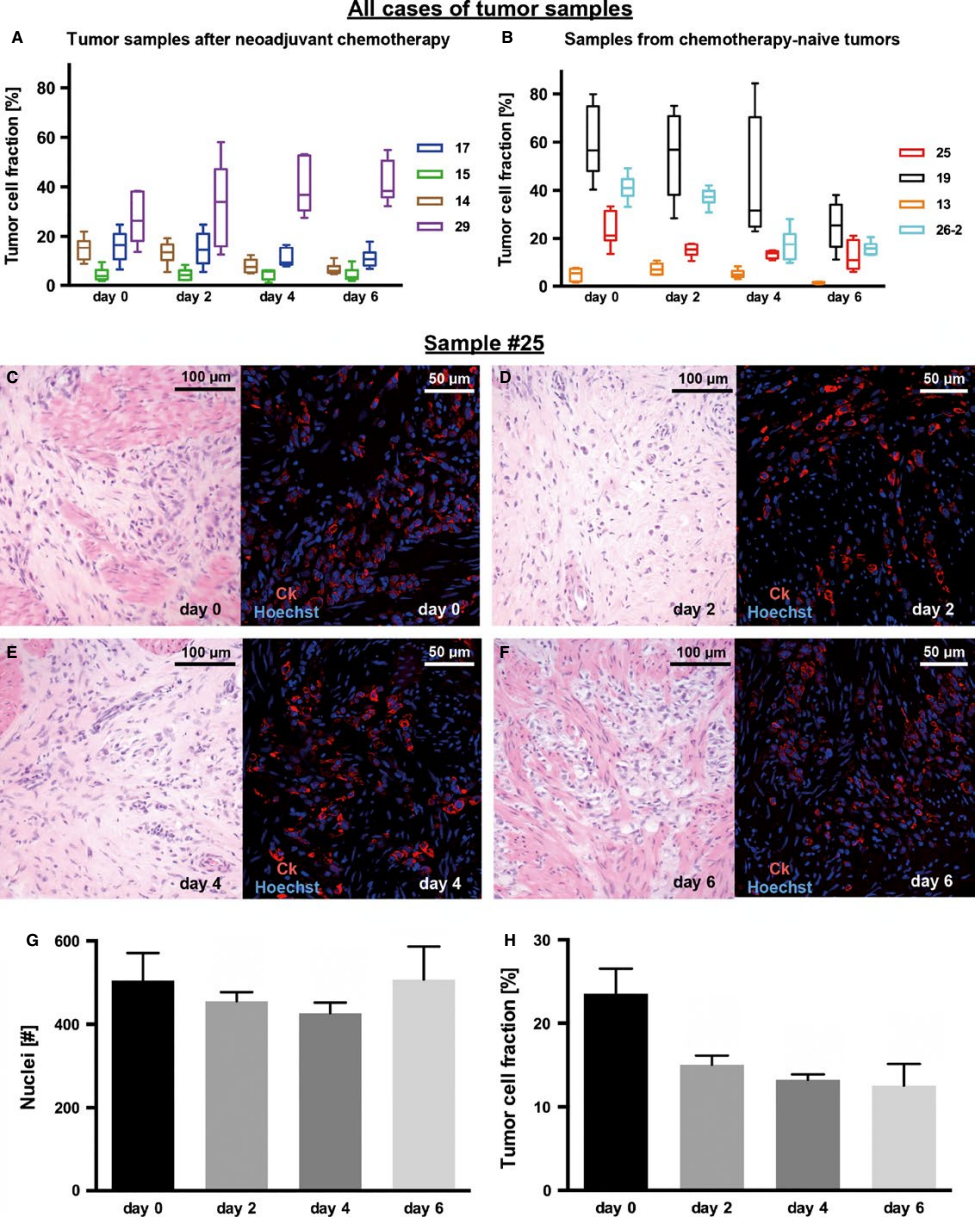
Quantification of total cell number and tumor cellularity. Panels (A–B) show tumor cellularity of all analyzed cases, while panels (C–H) display observations in one selected case (sample #25). (A) Four patients had received neoadjuvant chemotherapy prior to the sampling of tumor for slice cultivation and (B) four patients had not received any neoadjuvant treatment. In five cases (13, 15, 17, 25, 29), a stable tumor cell fraction until day 6 was observed. Three cases (14, 19, 26) revealed a distinct decrease in tumor cellularity between day 2 and day 4. (C–F) Slice cultures of sample #25 revealed a good preservation of tissue morphology and tumor cell integrity over 2, 4, and 6 days compared with day 0. Stromal cells were dominant in this tumor and the diffuse tumor pattern was difficult to distinguish in H&E stains. Quantification was therefore carried out on the basis of immunohistochemistry (right side). Tumor cells were detected with cytokeratin (CK) antibodies (red) which was combined with nuclear counterstaining (Hoechst 33342, blue). (G) Total cell number remained stable at all culture time points compared with day 0. (H) The tumor cell fraction decreased significantly in this particular case within the first 2 days of cultivation, but remained stable for the further culture period. Fluorescent microscopy, original magnification: 200× in C–F. ±SEM,* n *= 6.

Four patients had received neoadjuvant chemotherapy (Fig. 3A) and four patients were chemonaive at the time point of tissue sampling (Fig. 3B). A stable tumor cell fraction until day 6 was observed in five cases. Three cases revealed a distinct decrease in tumor cells between day 2 and day 4 (Fig. 3A and B).

Regarding the total cell count at each culture time point, the majority of cases remained stable until day 6 (data not shown). Neither neoadjuvant chemotherapy nor the tumor cell fraction measured at day 0 had a major impact on the stability of the tissue slices during cultivation (Fig. 3A and B). Figure 3G–H illustrates tumor cell quantification in one sample (#25).

**Figure 4:**
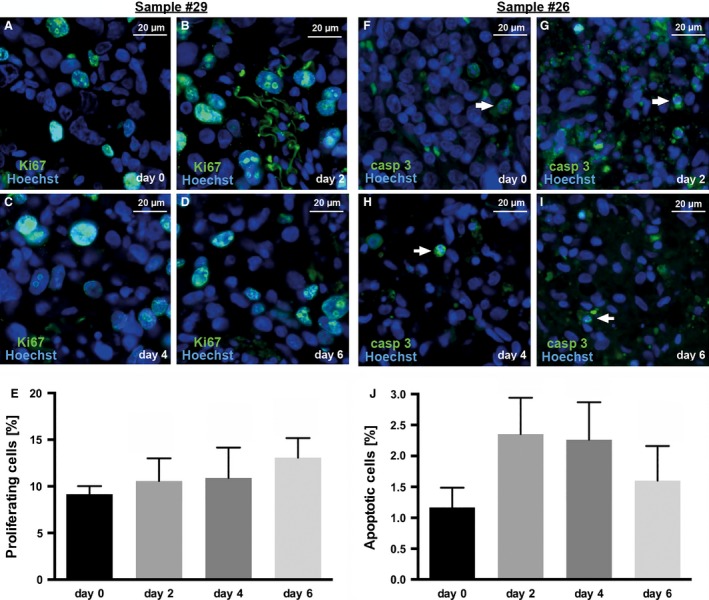
Proliferation (A–E) and apoptosis (F–J) indices in slice cultures of one human AEG (A–E) and one GC (F–J) specimen over a 6‐day culture period without cytotoxic drug exposure. (A–D) Proliferating cells were visualized using Ki‐67 staining (green) and (F–I) apoptotic cells were visualized using caspase‐3 staining (green) and were combined with nuclear counterstaining (Hoechst 33342; blue). (E) The proliferation indices did not show a decrease during the culture period proving a stable cell viability of cultured slices. (F–I) Basal apoptosis (white arrows) was observed at day 0 and every culture time point. (J) No significant increase in apoptotic cells was detected during the culture period. Fluorescent microscopy, original magnification: 400× in A–I, ±SEM,* n* = 3.

Figure 4 shows proliferation and apoptosis indices of two different tumor samples using anti‐Ki‐67 and anti‐cleaved caspase‐3 immunohistochemistry.

### Cytotoxic treatment in vitro

**Figure 5:**
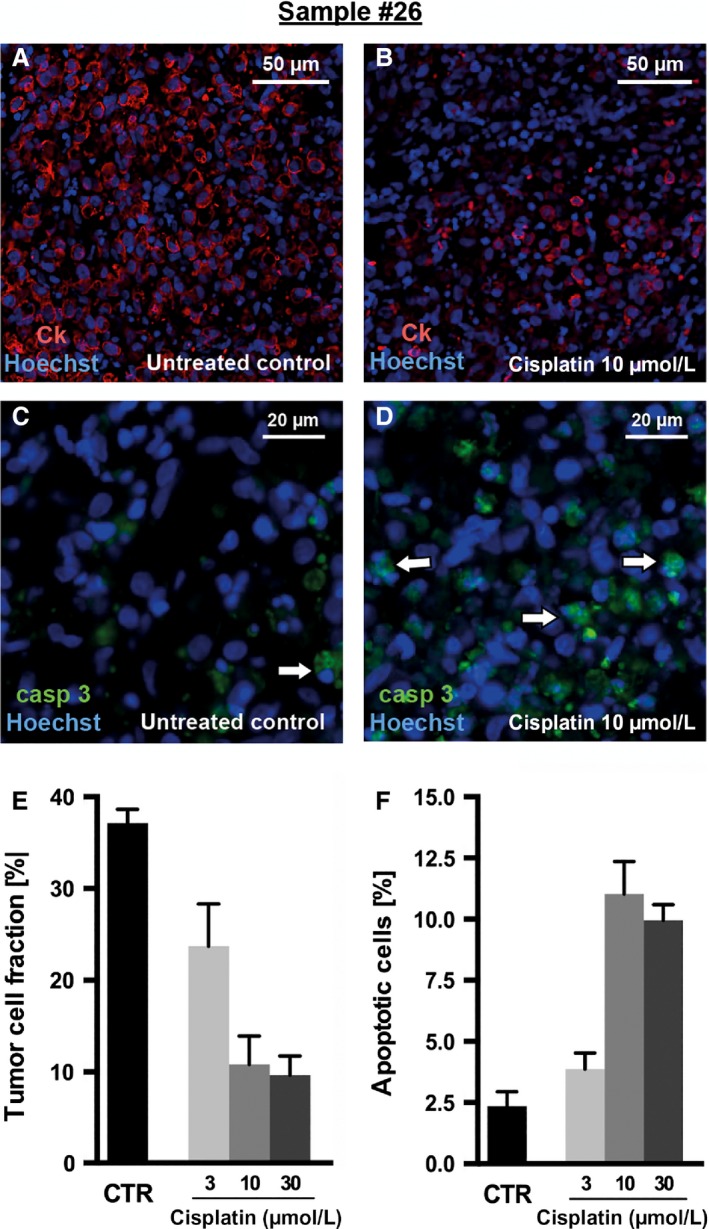
Effects of cytotoxic drug exposure in GC slice cultures. Slices from sample #26 were incubated with cisplatin and 5‐FU over 2 days and were then fixed and processed to paraffin sections (7 *μ*m). (A–B) Tumor cells were visualized using cytokeratin (CK) staining (red); (C–D) apoptotic cells were visualized using caspase‐3 staining (green). Both were combined with nuclear counterstaining (Hoechst 33342; blue) for quantitative analysis. (A) Untreated controls showed a dense and compact tumor cellularity, whereas (B) treated slices revealed a massive loss of tumor cells. (C) Compared to a minimal number of apoptotic cells (white arrows) in the untreated control, (D) treatment led to increased apoptosis. The loss of tumor cells after chemotherapy and the increase in apoptotic cells are illustrated in the bar graphs (E, F). Fluorescent microscopy, original magnification: 200× in A–B and 400× in C–D, ±SEM,* n *= 6 (CK), *n* = 3 (caspase‐3).

After cytotoxic treatment, H&E‐stained sections displayed a higher number of fragmented nuclei compared with untreated controls (not shown). Figure 5 illustrates the cytotoxic drug effects in one particular case. A decrease in tumor cellularity (Fig. 5A and B) and an increase in apoptotic processes (Fig. 5C and D) were observed upon treatment with cisplatin. This effect could be quantified (Fig. 5E and F). However, rather inhomogeneous results of apoptosis indices and higher standard errors were observed in some other cases. Therefore, we decided to use cytokeratin staining reflecting tumor cellularity for further analysis because the results obtained for this parameter were most consistent.

**Figure 6:**
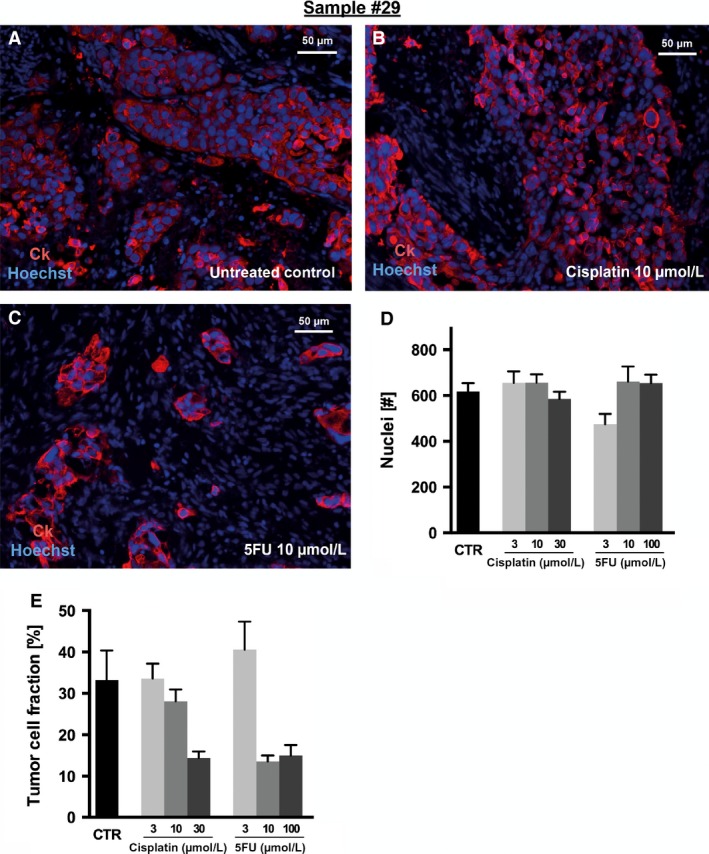
Tumor cellularity after cytotoxic drug exposure of AEG slice cultures. Slices from sample #29 were incubated with cisplatin and 5‐FU over 2 days and were then fixed and processed to paraffin sections (7 *μ*m). (A–C) Tumor cells were visualized using cytokeratin (CK) staining (red) and were combined with nuclear counterstaining (Hoechst 33,342; blue) for quantitative analysis. (A) Untreated controls revealed a dense and compact tumor cellularity which was not altered by exposure to (B) cisplatin 10 *μ*mol/L, whereas (C) 5‐FU 10 *μ*mol/L led to a massive loss of tumor cells. (D) The total number of nuclei was not reduced, neither by cisplatin nor by 5‐FU. (E) Tumor cellularity decreased upon treatment with higher concentrations of cisplatin (30 *μ*mol/L), while 3 or 10 *μ*mol/L cisplatin showed no effect on tumor cellularity. (E) A reduction in tumor cellularity after 5‐FU treatment was observed at a concentration of 10 *μ*mol/L or higher but not at the lower concentration. Fluorescent microscopy, original magnification: 200× in A–C, ±SEM,* n* = 6.

Figure 6 shows one case with a tumor cell fraction above 10% at day 0. The untreated control revealed a dense and compact tumor cellularity. This remained unchanged upon treatment with cisplatin 10*μ*mol/L, while treatment with 5‐FU 10 *μ*mol/L led to a massive decrease in tumor cellularity (Fig. 6A and C).

Three patient samples revealed a low tumor cellularity (4–8% of total cells) at day 0 (stroma predominant tumors). For these cases, we observed that the analysis of chemotherapy effects in vitro is more difficult and leads to greater standard errors (data not shown).

Regarding the other five patient samples (with an initial tumor cellularity above 10%), more reliable values on the >50% reduction in tumor cellularity after cisplatin or 5‐FU exposure were observed. Notably, dose‐dependent decreases of cellularities were measured, indicating the potential of this system for the assessment of individual drug sensitivities (Fig. S2).

Four samples were exposed to chemotherapy over 4 days to assess if a prolonged exposure to cytotoxic drugs leads to more loss of tumor cellularity or increased apoptosis. But systematic differences between both incubation periods were not observed (data not shown) indicating that the shorter incubation time is sufficient for assessing chemosensitivity.

## Discussion

To the best of our knowledge, this is the first description of the successful establishment of ex vivo organotypic slice cultures of human gastric and esophagogastric junction cancers. Importantly, we can also show that cytotoxic drug effects like changes in tumor cellularity, proliferation, and apoptosis can be assessed in this model making this new technique an interesting platform for the assessment of individual drug response.

Inter‐ and intra‐tumoral heterogeneity which are hallmarks of GC and AEG underscore the need for the development of more precise and personalized treatment 20. The moderate response rates to standard chemotherapy and the critical prognosis even after curative resection demonstrate the need for more efficacious treatment strategies. This could be reinforced by new translational research models which provide better treatment responses prediction for perioperative treatment 21. As a prerequisite, new laboratory‐based ex vivo cancer models should accurately reflect the molecular composition and heterogeneity of the tumor, including the stroma, being essential for the evolution of treatment resistance. In addition, the system should allow for the rapid assessment of cytotoxic drug effects in order to be ready for use in clinical practice.

Current ex vivo cancer models mainly rely on human primary cell lines, cell line‐derived or patient‐derived tumor xenografts. In contrast to our slice culture model, primary cell lines are artificial due to the selection of fast‐growing tumor cells during cultivation and the absence of any stroma. As a consequence, tumor–stroma interactions as well as heterogeneity are underrepresented 22, 23. In tumor xenograft models, a tumor microenvironment develops, but within murine stroma and in immunocompromised animals. This complicates the translation of findings to the situation in vivo. In addition, establishment of patient‐derived xenografts is time consuming and expensive and may thus not be an ideal tool for use in routine clinical practice. As previously shown in our laboratory, slice cultures of human glioblastoma and epithelial head and neck cancers could be successfully established. We experienced that each tumor entity needs careful and distinct investigation regarding the preparation process and culture conditions 16, 17, 18.

In our study, we found that slices of GC and AEG specimens can be cultured ex vivo over 6 days. We observed drug‐induced tissue alterations and reduction in tumor cellularity after exposure to varying doses of cisplatin or 5‐FU. Relevant cellular processes, that is, tumor and stromal proliferation as well as apoptosis, were observed. In our view, the advantage of this new system is the preservation of the natural epithelial–stromal architecture and the original tumor tissue morphology. The cultivation period provides sufficient time for investigating response to cytotoxic treatment.

A future challenge will be the establishment of slices derived from endoscopic biopsies which are needed to provide a platform for testing drug response prior to treating a patient in the neoadjuvant setting. Moreover, correlation of ex vivo findings with clinical response and outcome must be examined. Besides, specific molecular characteristics for response or resistance to chemotherapy should be unraveled in the next steps.

In conclusion, slice cultures derived from human gastric and esophagogastric adenocarcinoma have been successfully established and may become an attractive tool for drug response prediction as well as an interesting research model for investigating tumor heterogeneity and molecular features of drug resistance.

## Conflict of Interest

Florian Lordick received research support from GlaxoSmithKline (GSK), Fresenius Biotech, and Merck‐Serono (via his institution). He received travel support to scientific meetings from Amgen, Merck‐Serono, Roche, and Taiho. He acted as an advisor or speaker (without accepting personal payments) for Amgen, Boston Biomedical, Lilly, Roche, and Taiho. All other authors have no conflicts to disclose.

## Supporting information


**Figure S1.** Characteristics of slice cultures.

**Figure S2.** Tumor cellularity after cytotoxic drug exposure (all cases).

**Table S1.** Patient and treatment characteristics of all samples used for slice culture experiments.
