# Supplementary figures and images for: Organotypic slice cultures of human gastric and esophagogastric junction cancer
Source: Cancer Med. 2016 Apr 12;5(7):1444–53. doi: 10.1002/cam4.720 (PMC4944870; doi:10.1002/cam4.720)

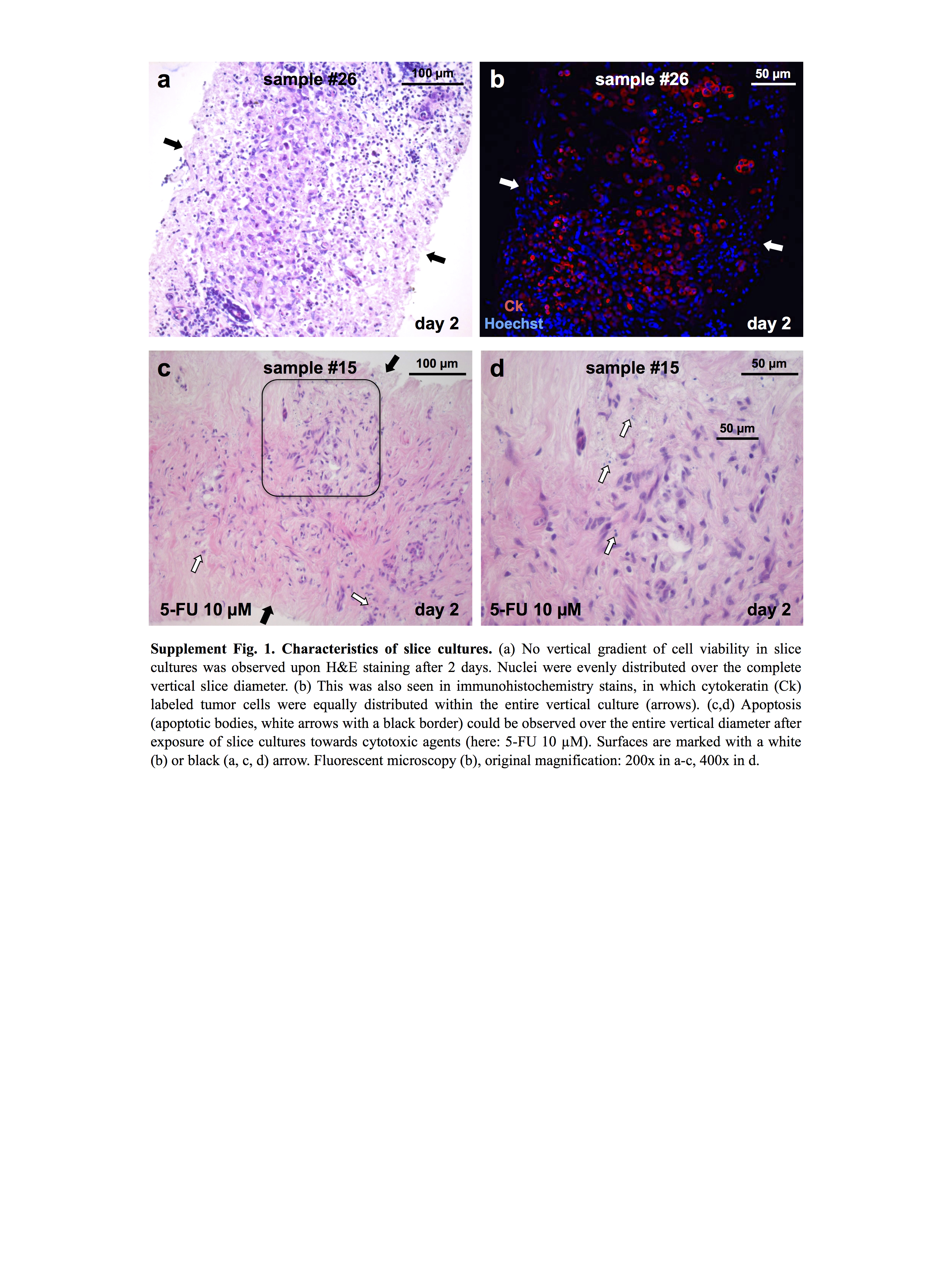

Supplement: Supplementary file 1 — Figure S1. Characteristics of slice cultures. [file CAM4-5-1444-s001.tiff]

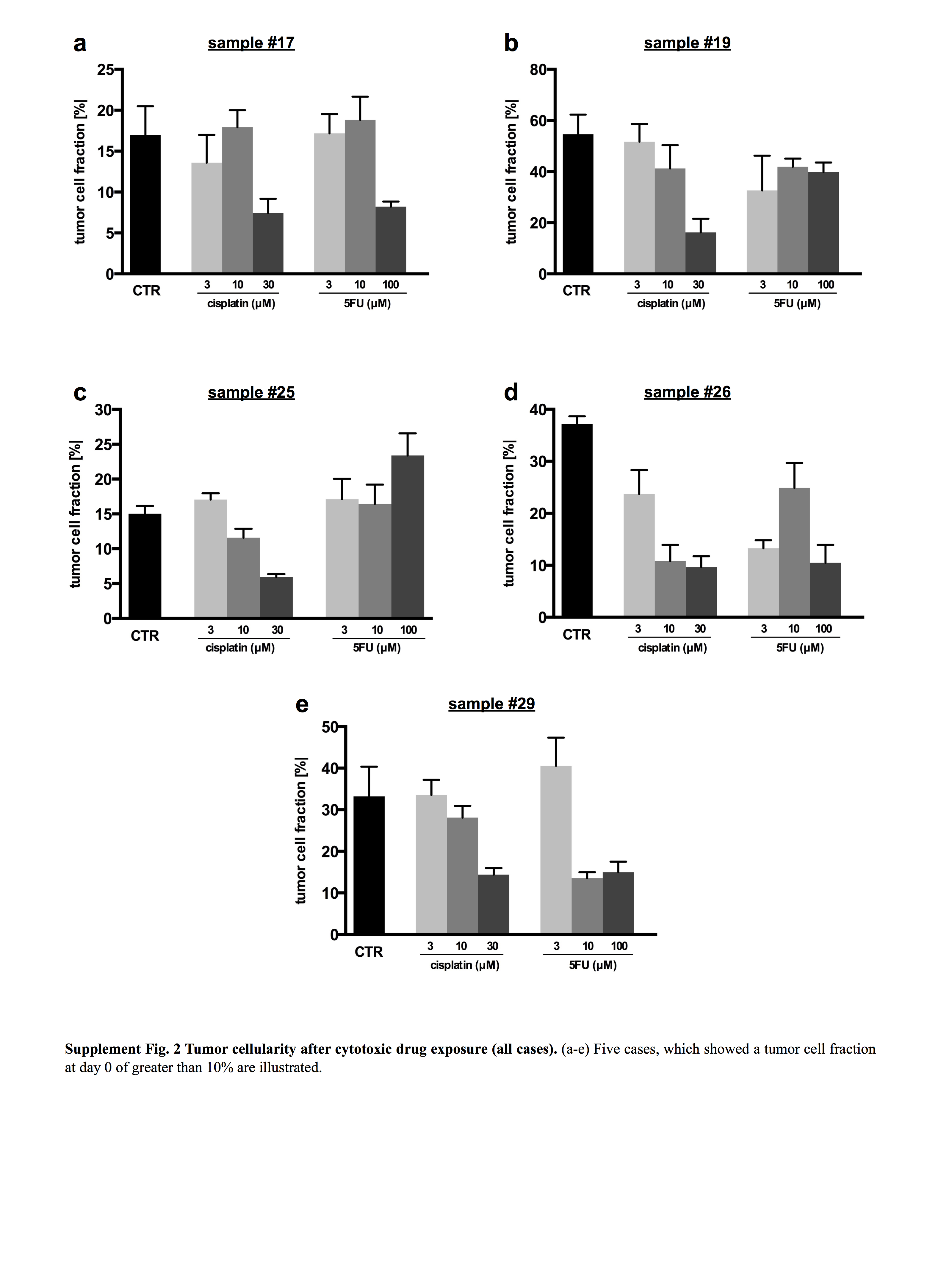

Supplement: Supplementary file 2 — Figure S2. Tumor cellularity after cytotoxic drug exposure (all cases). [file CAM4-5-1444-s002.tiff]
