# Supplementary material for: Organotypic slice cultures of human gastric and esophagogastric junction cancer
Source: Cancer Med. 2016 Apr 12;5(7):1444–53. doi: 10.1002/cam4.720 (PMC4944870; doi:10.1002/cam4.720)
Supplement: Supplementary file 3 — Table S1. Patient and treatment characteristics of all samples used for slice culture experiments. [file CAM4-5-1444-s003.docx]

| **sample number** | **origin** | **neoadjuvant chemotherapy** | **time until cultivation** | **culture period** | **displayed** | **histology and grading** |
| --- | --- | --- | --- | --- | --- | --- |
| 13 | GC | no | 6h | 1d, 2d, 4d, 6d | Analysis (Fig. 3b) | adenocarcinoma, diffuse type, G3 |
| 14 | GC | yes | 4h | 2d, 4d, 6d | Analysis (Fig. 3a) | adenocarcinoma, intestinal type, G3 |
| 15 | GC | yes | 8h | 2d, 4d, 6d | Analysis (Fig. 3a) | adenocarcinoma, diffuse type, G3 |
| 17 | AEG | yes | 2h | 2d, 4d, 6d | Analysis (Fig. 3a) | adenocarcinoma, diffuse type, G3 |
| 19 | GC | no | 5h | 2d, 4d, 6d | Analysis (Fig. 1 a-d, Fig. 2 a-d; Fig. 3b) | adenocarcinoma, intestinal type, G2 |
| 25 | GC | no | 6h | 2d, 4d, 6d | Analysis (Fig. 3 c-f, b) | adenocarcinoma, diffuse type, G3 |
| 26 | GC | no | 4h | 2d, 4d, 6d | Analysis (Fig. 2 e-h; Fig. 3b; Fig. 4 f-j; Fig. 5 a-j) | adenocarcinoma, diffuse type, G3 |
| 29 | AEG | yes | 6h | 2d, 4d, 6d | Analysis (Fig. 1 e-g, Fig. 3a, Fig. 4 a-e, Fig. 6) | adenocarcinoma, diffuse type, G3 |

**Supplement Table 1: Patient and treatment characteristics of all samples used for slice culture experiments.**
